# Supplementary material for: Trials of the Automated Particle Counter for laboratory rearing of mosquito larvae
Source: PLoS One. 2020 Nov 10;15(11):e0241492. doi: 10.1371/journal.pone.0241492 (PMC7654806; doi:10.1371/journal.pone.0241492)
Supplement: S1 Fig — In order to eliminate egg shells, An. gambiae embryos were placed on filter papers raised slightly above the water level on cloth sponge disc platforms. After hatching, larvae wriggle off the paper into the water and the filter paper can be lifted out, completely removing the shells. The larvae were then collected on a fine screen and the food particles decanted away with two or three rounds of slow pouring. It is important to use a sponge cloth that is smooth because textured cloths allow the larvae to collect on top of the sponge rather than wriggling into the water. (DOCX) [file pone.0241492.s001.docx]

| 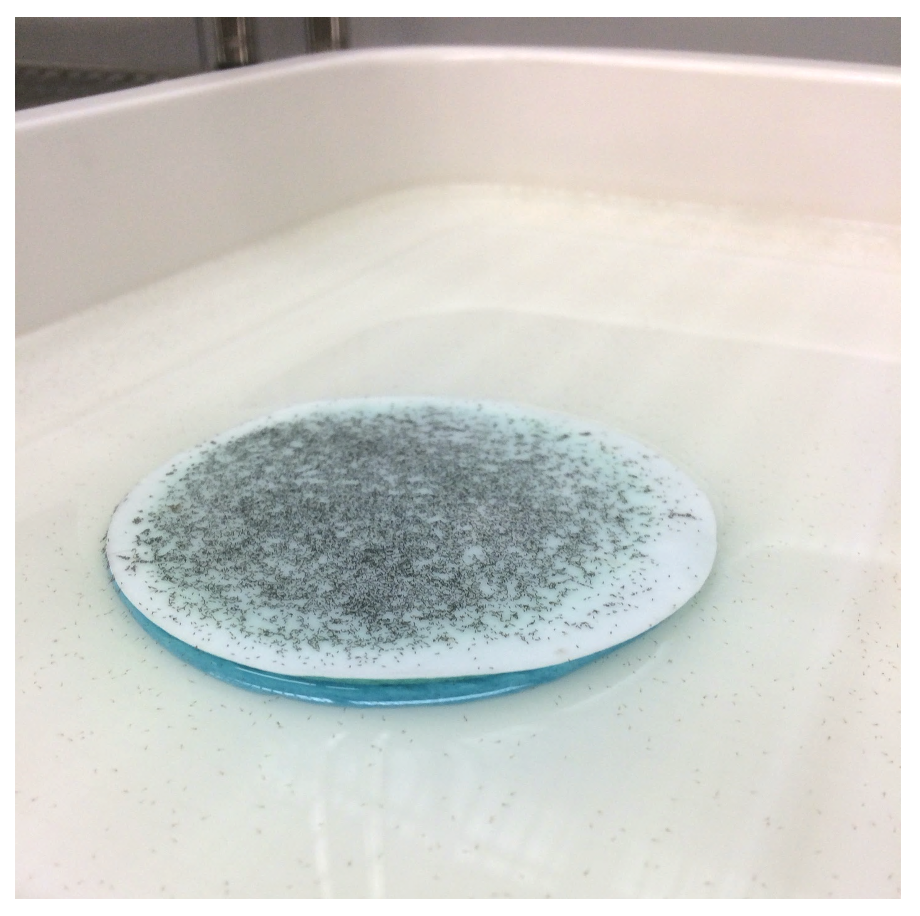 |
| --- |
| **S1 Fig. Egg hatching arrangement**. In order to eliminate egg shells, *An. gambiae* embryos were placed on filter papers raised slightly above the water level on cloth sponge disc platforms. After hatching, larvae wriggle off the paper into the water and the filter paper can be lifted out, completely removing the shells. The larvae were then collected on a fine screen and the food particles decanted away with two or three rounds of slow pouring. It is important to use a sponge cloth that is smooth because textured cloths allow the larvae to collect on top of the sponge rather than wriggling into the water. |
