# Supplementary material for: Trials of the Automated Particle Counter for laboratory rearing of mosquito larvae
Source: PLoS One. 2020 Nov 10;15(11):e0241492. doi: 10.1371/journal.pone.0241492 (PMC7654806; doi:10.1371/journal.pone.0241492)
Supplement: S2 Fig — Larvae were dispensed into small plastic cups and were transferred using a vacuum filter apparatus onto 15 cm diameter filter paper discs with the dispensing run sample identification number and target number written on it before being photographed. (DOCX) [file pone.0241492.s002.docx]

| 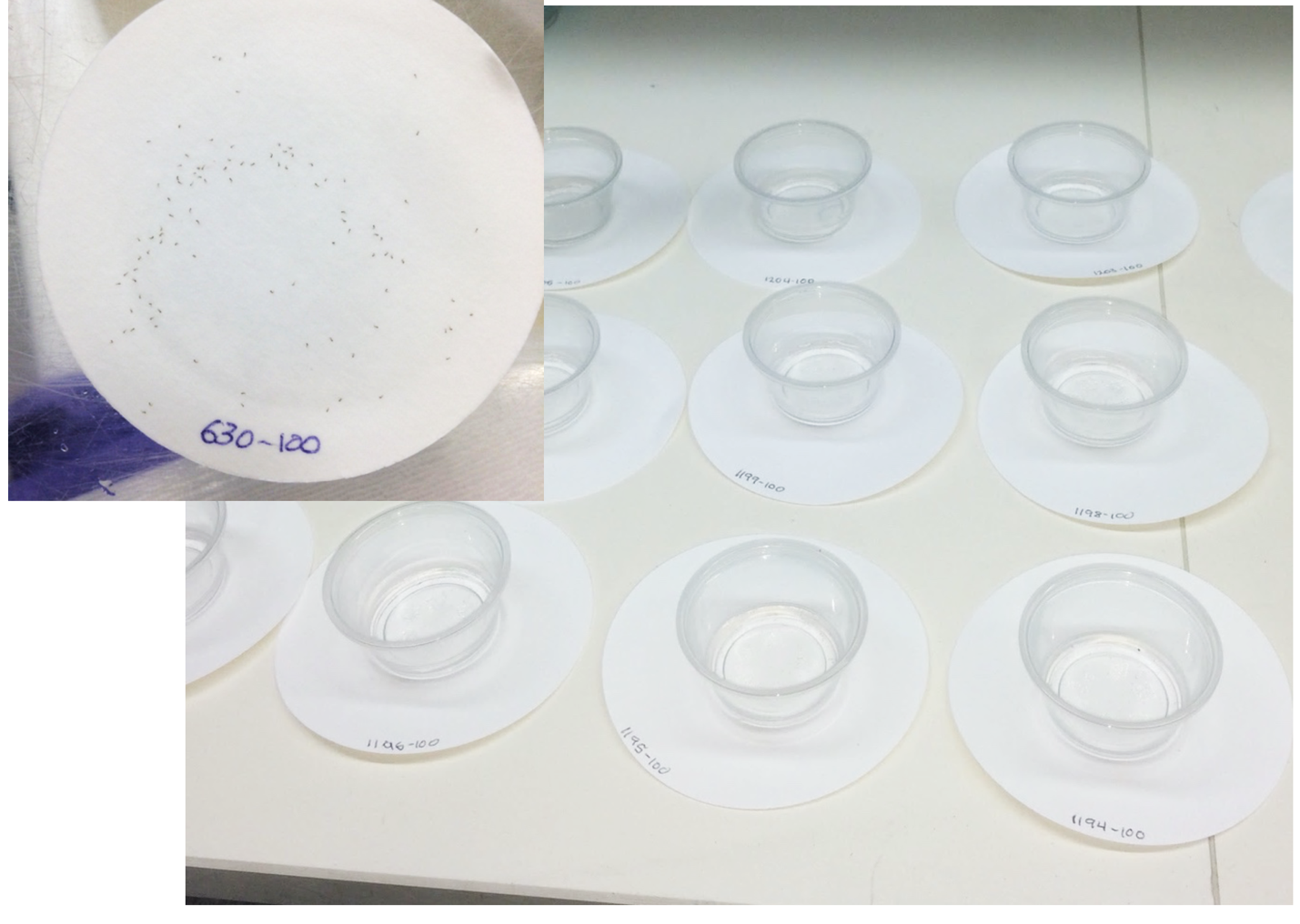 |
| --- |
| **S2 Fig. Dispensed larvae**. Larvae were dispensed into small plastic cups and were transferred using a vacuum filter apparatus onto 15 cm diameter filter paper discs with the dispensing run sample identification number and target number written on it before being photographed. |
