# Supplementary material for: Trials of the Automated Particle Counter for laboratory rearing of mosquito larvae
Source: PLoS One. 2020 Nov 10;15(11):e0241492. doi: 10.1371/journal.pone.0241492 (PMC7654806; doi:10.1371/journal.pone.0241492)
Supplement: S3 Fig — This capacity allows the user to recall the specific conditions that were used. The data is output in a comma delimited file (CSV) format and can be downloaded and stored in e.g. Microsoft Excel. These data were observed using the web browser interface via a WiFi connection. (DOCX) [file pone.0241492.s003.docx]

| 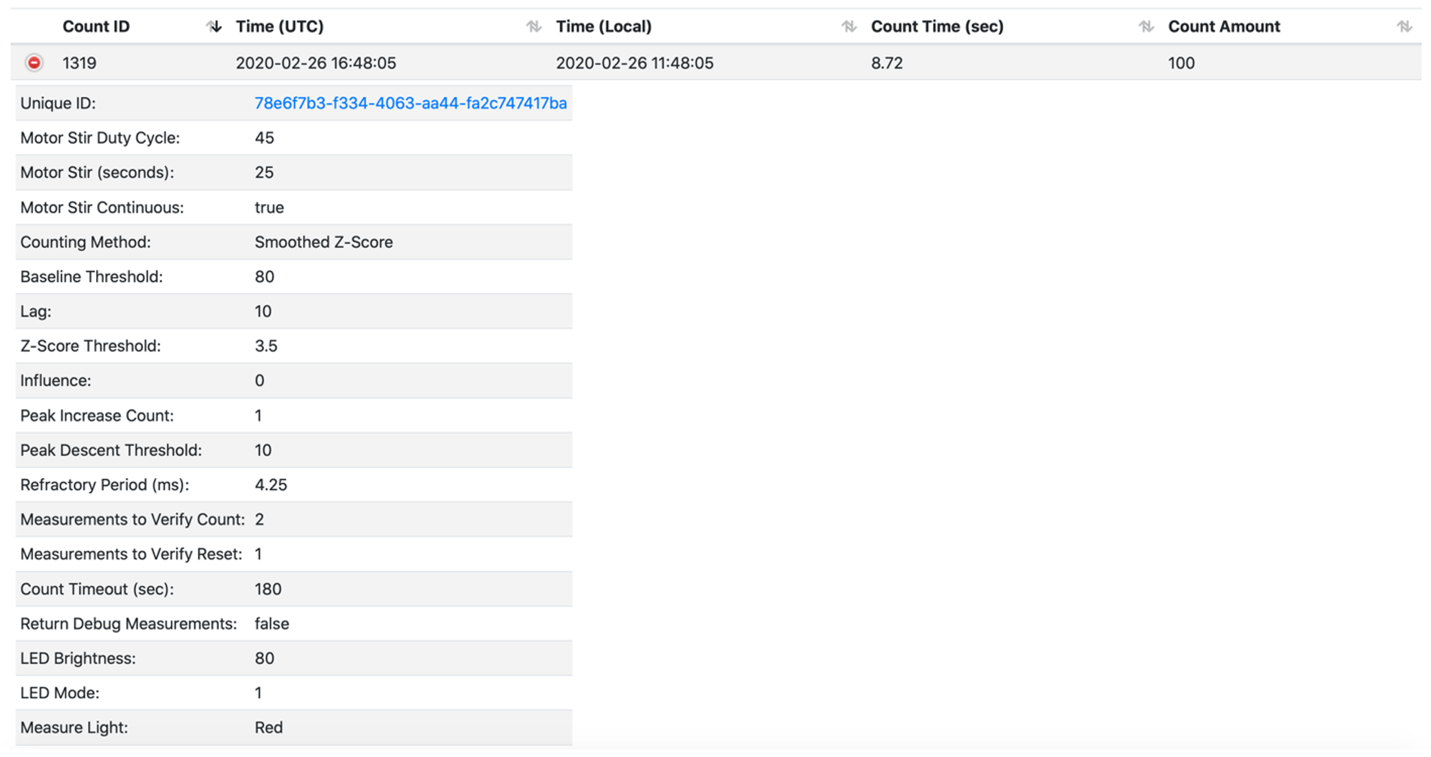 |
| --- |
| **S3 Fig. Details captured for the individual runs.** This capacity allows the user to recall the specific conditions that were used. The data is output in a comma delimited file (CSV) format and can be downloaded and stored in e.g. Microsoft Excel. These data were observed using the web browser interface via a WiFi connection. |
