# Supplementary material for: Trials of the Automated Particle Counter for laboratory rearing of mosquito larvae
Source: PLoS One. 2020 Nov 10;15(11):e0241492. doi: 10.1371/journal.pone.0241492 (PMC7654806; doi:10.1371/journal.pone.0241492)
Supplement: S4 Fig — The peaks (lower section) are the amount of light sensed below the baseline. The method being used here is the ‘Smoothed Z-Score’. Blue bars and points indicate all recorded absorbances above the Z score, whereas red points and bars indicate time points at which a ‘count’ is tabulated. Toward the right is a peak in which two larvae were counted that passed the detector in close succession. The sensor data can be collected in a detailed “diagnostic mode” or normal mode; the latter allows faster processing while the former can be used to develop parameters for the settings using the specific samples to be dispensed but requires more processing time and is not practical for dispensing runs of > 10 particles. Both modes create records of the settings for each dispensing run. While diagnostic mode data is not necessary for routine use, it can be helpful for testing the device as we did here and for establishing the proper conditions for routine operation. These can be visualized on a computer using a Wifi connection to the APC and can be transferred in CSV file format file for import into e.g. Microsoft Excel. (DOCX) [file pone.0241492.s004.docx]

| 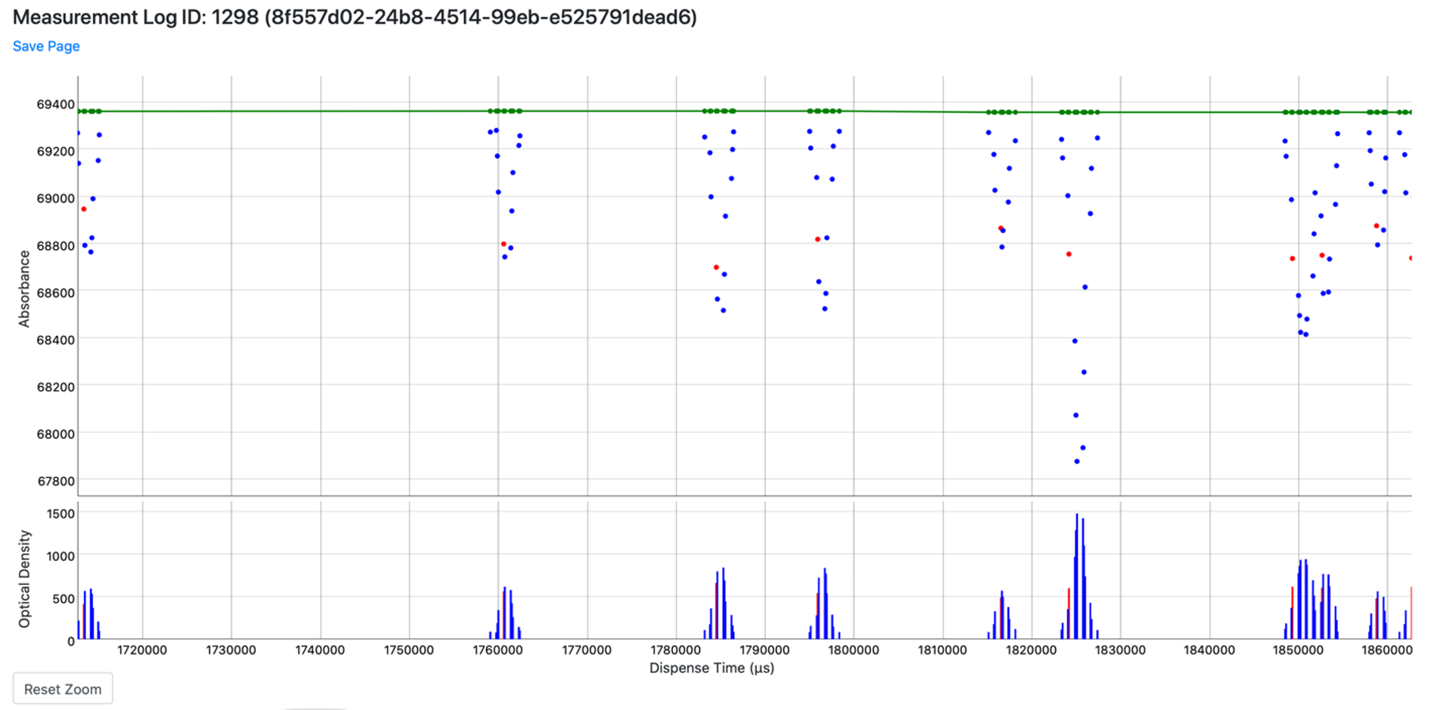 |
| --- |
| **S4 Fig. Graph of data collected in diagnostic mode.** The peaks (lower section) are the amount of light sensed below the baseline. The method being used here is the ‘smoothed Z score’. Blue bars and points indicate all recorded absorbances above the Z score, whereas red points and bars indicate time points at which a ‘count’ is tabulated. Toward the right is a peak in which two larvae were counted that passed the detector in close succession.  The sensor data can be collected in a detailed “diagnostic mode” or normal mode; the latter allows faster processing while the former can be used to develop parameters for the settings using the specific samples to be dispensed but requires more processing time and is not practical for dispensing runs of > 10 particles. Both modes create records of the settings for each dispensing run. While diagnostic mode data is not necessary for routine use, it can be helpful for testing the device as we did here and for establishing the proper conditions for routine operation. These can be visualized on a computer using a Wifi connection to the APC and can be transferred in CSV file format file for import into e.g. Microsoft Excel. |
