# Supplementary material for: Trials of the Automated Particle Counter for laboratory rearing of mosquito larvae
Source: PLoS One. 2020 Nov 10;15(11):e0241492. doi: 10.1371/journal.pone.0241492 (PMC7654806; doi:10.1371/journal.pone.0241492)
Supplement: Attachment — Submitted filename: Response to Reviewers.docx [file pone.0241492.s005.docx]

Response to Reviewers:

PONE-D-20-24168
***Trials of the Automated Particle Counter for laboratory rearing of mosquito larvae***
PLOS ONE

Benedict, MQ *et al.*

Dear Editor,

Thank you for the opportunity to prepare a revised manuscript including our detailed responses to the recommendations and comments of the reviewers and editorial staff.

In addition to the marked-up Word DOC file of the manuscript, we are providing explanatory information below.

Sincerely, on behalf of the authors,

Mark Q. Benedict

Journal Requirements:

When submitting your revision, we need you to address these additional requirements.

Comment:

1. Please ensure that your manuscript meets PLOS ONE's style requirements, including those for file naming. The PLOS ONE style templates can be found at

<https://journals.plos.org/plosone/s/file?id=wjVg/PLOSOne_formatting_sample_main_body.pdf> and

<https://journals.plos.org/plosone/s/file?id=ba62/PLOSOne_formatting_sample_title_authors_affiliations.pdf>

Response:

We have checked the formatting against the recommendation and have altered heading fonts, author affiliation and mane formatting and file names particularly.

Comment:

2. We note that you have stated that you will provide repository information for your data at acceptance. Should your manuscript be accepted for publication, we will hold it until you provide the relevant accession numbers or DOIs necessary to access your data. If you wish to make changes to your Data Availability statement, please describe these changes in your cover letter and we will update your Data Availability statement to reflect the information you provide.

Response:

We will provide a URL / DOI upon acceptance.

Comment:

3. Thank you for stating the following in the Acknowledgments Section of your manuscript:

[Funding Disclosure Statement: MQB, PB, CMH, RDR and EIA are paid employees of Target Malaria, a project that receives core funding from the Bill & Melinda Gates Foundation and from the Open Philanthropy Project Fund, an advised fund of Silicon Valley Community Foundation to the Target Malaria project.]

We note that you have provided funding information that is not currently declared in your Funding Statement. However, funding information should not appear in the Acknowledgments section or other areas of your manuscript. We will only publish funding information present in the Funding Statement section of the online submission form.

Please remove any funding-related text from the manuscript and let us know how you would like to update your Funding Statement. Currently, your Funding Statement reads as follows:

 [The funds for this study were awarded to the Foundation for the Centers for Disease Control and Prevention.]

Response:

This is actually one of the most confusing aspects of submissions to PLoS journals!

We have removed the statement above from the Acknowledgments

Would you please **revise the Funding Statement** to read as follows:

“MQB, PB, CMH, RDR and EIA are paid employees of Target Malaria, a project that receives core funding from the Bill & Melinda Gates Foundation and from the Open Philanthropy Project Fund, an advised fund of Silicon Valley Community Foundation to the Target Malaria project. The funds for this study were awarded to the Foundation for the Centers for Disease Control and Prevention. The funders had no role in study design, data collection and analysis or decision to publish.”

Comment:

4. Please upload a copy of Figure 6, to which you refer in your text on page 14. If the figure is no longer to be included as part of the submission please remove all reference to it within the text.

Response:

Thank you for catching this. During drafting, we combined some individual figures into composites of two and missed this change in the reference. We have corrected the reference to the figure which should have been “4b” rather than “Fig 6”.

Reviewer #1: Overview
In this paper draft, Benedict et al reviews the performance of a commercially available counter for L1 mosquito larvae. The authors give a broad overview of the device and parameters that can be adjusted when operating the device. The authors test the accuracy of the counter using two morphological different mosquito species and determined that the counter is accurate enough to use for routine rearing in a laboratory setting. The paper is well written, in clear English and easy to follow. The experimental design is sufficiently robust to support the results obtained and the discussion is supported by the observed results. Within this context, I think the manuscript meets the criteria for publication.

I do, however, have some concerns with the scope of testing and whether the authors have done enough to provide potential users of the system with the data required to make an informed choice on the whether the unit would provide a productivity improvement. I have some suggestions for additional testing that would give the manuscript more relevance to a wider audience.

Major Questions/Suggestions

Comment 1:

Can the authors give some details on what the other algorithms are that are available and why one the Smoothed Z score was selected for testing?

Response 1:

Yes. We have modified the text (shown in italics) as follows to explain in more detail what the options were and why we chose the Smoothed Z Score.

“The APC senses changes in light intensity in real time, analyzes them and accumulates the counts using one of several algorithms chosen and parameterized by the user. *The device is equipped with three algorithms: 1) ‘Threshold Reset’ which requires a user specified number of measurements above and below a baseline threshold to identify separate particles; 2) ‘Refractory Period’ is similar to ‘Threshold Reset’ but requires passage of a user specified amount of time between particles rather than a number of measurements. Both of these seem useful for well-separated particles but we believe would be poor at identifying overlapping particles*. 3), We tested the ‘Smoothed Z-Score.

We also replaced “Smoothed Z score” with “Smoothed Z-Score” throughout the manuscript to match the manufacturer’s style of naming this algorithm.

Comment 2:
What is the productivity break point for small scale rearing using the counter vs manual aliquoting? I think the authors should provide some additional data comparing the speed and accuracy of the counter against a human operator. At which point does it become more productive to use the counter vs just manual aliquoting? For instance, if I am rearing ten trays at a density of 250 larvae per week, does it make more sense to use the counter or should I stick with a technician?

Response 2:

A very good question that will indeed be asked by many readers, so we asked the staff to provide some estimates for this purpose by measuring the amount of time required for manual dispensing vs the APC. Their observations were enlightening. We have added the following to the Discussion to give a rough estimate of the break-even point.

“*Because the APC requires setup and breakdown time whereas manual counting can be initiated with almost none of these, we estimated the amount of time required for either activity in a small preliminary trial. The time required to dispense 20 trays of 250 with the APC was 21 min 10 sec which includes 3 min 39 sec of setup and breakdown time. Manual counting of nine trays of 250 larvae by three people counting three trays each required an average of 3 min 18 sec per tray (range 2 min 12 sec – 4 min 10 sec) with essentially no setup and breakdown time. Because setup and breakdown time is fixed, regardless of the number of trays dispensed, dispensing larger numbers of trays using the APC reduces the time per tray. However, if one is counting even two trays, it requires slightly less time to perform the dispensing using the APC (5 min 24 sec) over manual counting (6 min 36 sec). Simplistically, the break-even point to choose between the two methods is two trays, but the directness of manual counting will possibly cause most users to use the APC only for higher numbers of trays.*”

We also changed our final list of recommendations to reflect this small trial and indicate that the APC is most useful for 3 or more trays.

Comment 3:

Applicability for large scale rearing? Typically, L1 counters become much more attractive options when the scale of rearing increases. For large scale rearing facilities, manual aliquoting of L1 larvae is not feasible and I think it is important to demonstrate that the counter is still accurate when aliquoting larger (>1000) batches of larvae. In addition, the speed of operation is just as crucial, and the authors should include a comparison table to show the total time required to aliquot a range of L1 larvae.

Response 3:
We have admittedly skirted around the issue of application scale but state in the title that these trials are for ‘laboratory rearing’ of mosquitoes which we believe is the best setting for its application. As the reviewer is clearly aware, the demands of mass-rearing are greater and can often be difficult to anticipate. Beside the capabilities of the COPAS, we don’t believe that any individual counting method is suitable for mass-rearing.

The data that the reviewer is asking for can be easily determined from the larva per second rates in Figure 3. Almost all of the measures we made were done at larva dispensing rates < 50 larvae per second (LPS). Therefore, the time required for different numbers is directly related to the LPS e.g. 250 will require 5 seconds at LPS = 50, and 25 seconds at LPS = 10, plus time between counts which is about 10 seconds for the machine to check the baseline.

While we tested the machine at counts of 100 for this manuscript, we had done some counts of 200 and found the manual counting times prohibitive. We have provided this preliminary data in the Discussion as follows:

“*Somewhat different dispensing error rates by the APC might be expected for larger numbers of larvae. In a small preliminary trial counting 200 An. gambiae larvae (n=19), we observed and average of 208 larvae (± 5.57) per dispensing. This contrasts with the under-dispensing observed with counts of 100. Therefore, users should determine the error rate that they experience with the specific numbers they dispense*.”

Minor questions/issues
Comment:

1. Line 233-235: Best Z-Score in Table 1 is 2.5 – why did the authors settle on 3.5? Can you provide a bit more context please?

Response:

We expanded upon the choice in the relevant section of the Results and modified it as follows (changes italicized):

“*As a result of seeing no difference in Z scores from 2.5-6* and the manufacturer’s recommendation, we routinely used a Z score of 3.5. *The manufacturer’s recommendation is based on a random error probability of 1/2128 data points (p = 0.00047).*”

Comment:
2. Line 285: There is no Fig 6.

Response:

Ouch. Thank you. When revising the drafts, we combined some figures and have corrected this based on your and the editor’s comments. Figure 6 should have been “4b”.

Reviewer #2: Overview
Benedict et al report trials of a flow-cytometer type device called the ‘Automated Particle Counter’ (APC) to count mosquito larvae. Through tests of a number of key parameters, including Z score, accuracy and precision and larvae survivorship, they demonstrated APC may serve as an useful tool to control mosquito larvae density, an essential step in mosquito mass rearing. These results will facilitate the current effort to improve the effectiveness and quality control of mosquito mass rearing for developing novel mosquito controls strategy. Below are some minor points.

Comment:

Figure 3 lists only the data of the measured value and LPS. How accuracy was calculated for the correlation test as indicated in the text (Line 250)? To be clear for the readers, the “accuracy” should be defined (degree of closeness of a measured value to its actual value?). It appears that data were enriched in low LPS and very few replicates was done at LPS over 60. Will this affect the conclusion?

Response:

Yes, the reviewer makes a good point. The underlying data was used to calculate accuracy (the definition of which by the reviewer we agree with) separately (Figure 4), however we did not determine accuracy in this

particular case except to the extent that we observe it’s unchanging (no significant slope) over the range tested.

Therefore, we changed the title of the figure to “Relationship between larval rate and number dispensed”

Comment:

Figure 4, “accuracy” and “precision” should be defined. Does this figure show only precision?

Line 268-290, what does the word “mode” mean? Why two different mode numbers were used for calculation of the accuracy?

Response:

Thank you for your sensitivity to these terms which are often inaccurately used. We have stated the definition we used throughout the manuscript in lines 88-90 as:

“Here, we determined the accuracy, as indicated by the difference between the number dispensed and the user-specified number, and the precision of the number dispensed by the APC, as reflected by the distribution of the data around the mean.”

Regarding the use of those terms in Figure 4, the distribution and mean that are discussed in the accompanying results are derived from this figure, but you are correct that there is no specific indication of the mean in that figure so we have changed the title to simply “Precision of dispensing” and the accuracy is presented in the text.

‘Mode’ is a statistical term meaning the most commonly observed number dispensed.

Comment:

It will be good to indicate the capacity of the APC in the discussion. For example, under an optimal condition, how much time is taken to calculate 1 million larvae or how many larvae can be dispensed in one hour?

Response:

The reviewer has touched upon an issue that the other reviewer noted as well. We will reiterate our response to that reviewer here:

“*We have admittedly skirted around the issue of application scale but state in the title that these trials are for ‘laboratory rearing’ of mosquitoes which we believe is the best setting for its application. As the reviewer is clearly aware, the demands of mass-rearing are greater and can often be difficult to anticipate. Beside the capabilities of the COPAS, we don’t believe that any individual counting method is suitable for mass-rearing*.”

The data that the reviewer is asking for can be easily determined from the larva per second rates in Figure 3. Almost all of the measures we made were done at larva dispensing rates < 50 larvae per second (LPS). Therefore, the time required for different numbers is directly related to the LPS e.g. 250 will require 5 seconds at LPS = 50, and 25 seconds at LPS = 10, plus time between counts which is about 10 seconds for the machine to check the baseline.

While we tested the machine at counts of 100 for this manuscript, we had done some counts of 200 and found the manual counting times prohibitive. We have provided this preliminary data in the Discussion as follows:

“*Somewhat different dispensing error rates by the APC might be expected for larger numbers of larvae. In a small preliminary trial counting 200 An. gambiae larvae (n=19), we observed and average of 208 larvae (± 5.57) per dispensing. This contrasts with the under-dispensing observed with counts of 100. Therefore, users should determine the error rate that they experience with the specific numbers they dispense*.”

Comment:
Please indicate whether and how the Z score can be manually set for Automated Particle Counter in the method section.

Response:

Yes, we have modified the text as follows (changes italicized):

“*By accessing the configuration menu on the touch-screen menu, the* user can select the number of standard deviations (Z score) needed to begin recording the intensity, the number of detections above the minimum to trigger a count and the number of declining values or the amount before initiating another count, which is useful to distinguish clustered individuals. *These settings are stored between uses of the device so that settings do not need to be entered each time.*
